# SREBP1-Dependent Metabolism as a Potential Target for Breast Cancer Risk Reduction

**DOI:** 10.3390/cancers17101664

**Published:** 2025-05-14

**Authors:** Atieh Hajirahimkhan, Kristy A. Brown, Susan E. Clare, Seema Ahsan Khan

**Affiliations:** 1 Division of Breast Surgery, Robert H. Lurie Comprehensive Cancer Center, Feinberg School of Medicine, Northwestern University, 303 E Superior, 4-220, Chicago, IL 60611, USA; susan.clare@northwestern.edu (S.E.C.); s-khan2@northwestern.edu (S.A.K.); 2 Department of Cell Biology and Physiology, University of Kansas Medical Center, Kansas City, KS 66160, USA; kbrown46@kumc.edu; 3 Cancer Prevention and Control Program, University of Kansas Cancer Center, Kansas City, KS 66160, USA

**Keywords:** breast cancer, inflammation, lipogenesis, metabolism, risk reduction, SREBP1

## Abstract

Breast cancer prevention remains a major challenge, despite the availability of endocrine therapies such as tamoxifen and aromatase inhibitors. Adverse effects, including arthralgia, are associated with poor uptake of endocrine therapy in the prevention setting, with more than 85% of risk-eligible women opting to discontinue their use or decline taking them a priori. Non-endocrine therapies with sufficient efficacy and minimal toxicities are needed. The master regulator of lipogenesis, sterol regulatory element binding protein 1 (SREBP1), has emerged as an important driver of breast cancer development, and, as such, a potential target for breast cancer interception. Following the review of studies published between 2015 and 2025, this manuscript summarizes the evidence for SREBP1 being a promising target to block breast cancer at multiple steps ranging from early to metastatic lesions. Our review identifies SREBP1 as a prognostic marker and supports disrupting SREBP1 activation, stabilization, and translocation to the nucleus as feasible targets for breast cancer prevention or interception. We conclude that SREBP1 is a promising non-endocrine target that can be exploited for breast cancer risk reduction in high-risk women. It needs to be further studied to define any potential side effects.

## Simple Summary

Most women at high risk for breast cancer avoid preventive endocrine treatments like tamoxifen due to side effects. Safer, non-hormonal options are urgently needed. This review highlights a key protein called SREBP1, which controls fat production in cells and plays a major role in breast cancer development. By reviewing studies from 2015 to 2025, we show that targeting SREBP1 may help prevent breast cancer from starting or spreading. SREBP1 also appears to predict how aggressive a breast tumor might be. Blocking its activity, movement in the cell, or stability may offer a new, well-tolerated strategy to reduce breast cancer risk. Further research is needed to explore the safety of its therapeutic and cancer preventive targeting.

## 1. Introduction

There are an estimated 10 million U.S. women, aged 35 to 79 years with a moderately increased risk of breast cancer (1.5–4-fold), for whom treatment with risk-reducing medications is known to be effective [1,2]. At present the only risk-reducing drugs available for breast cancer are aromatase inhibitors (AIs) and selective estrogen receptor modulators (SERMs). In randomized clinical trials (RCTs), these drugs can lower the incidence of estrogen receptor-positive (ER+) breast cancer by 50–65%, with a relative risk reduction of ~70% in women with atypical hyperplasia (AH) and lobular carcinoma in situ (LCIS) [1,3]. However, more than 85% of risk-eligible women decline them, mainly due to their acknowledged adverse effects: vasomotor symptoms, thrombogenesis/stroke, bone loss, and in the case of tamoxifen, an increased risk of endometrial cancer [3,4,5,6,7,8,9,10]. A recent RCT demonstrated that low-dose tamoxifen (5 mg daily) taken for three years reduced breast cancer incidence by 52% in women with preinvasive or atypical lesions, with fewer side effects than the conventional dose [11]. Nevertheless, the use of risk-reducing medications among risk-eligible women remains low at under 15% [12]. In addition, AIs and SERMs do not protect against estrogen receptor-negative (ER-) breast cancer. With the rapidly growing body of evidence on the role of metabolic dysregulations in breast carcinogenesis, inhibitors of PI3K-AKT signaling, already used in the treatment setting, have been explored for breast cancer prevention, but adverse effects such as hyperglycemia and diarrhea prohibit their application in the prevention setting [13]. Therefore, there is a clear need for alternative risk-reducing drugs; ideally, such drugs would have broader efficacy that extends to ER- breast cancer, minimal adverse effects, either acceptance by a majority of at-risk women or potential for precision prevention.

## 2. SREBP1—The Master Regulator of Lipogenic Metabolism

Sterol regulatory element-binding protein 1 (SREBP1), which regulates the synthesis of fatty acids and cholesterol by controlling the expression of genes necessary for these processes, is an extensively studied protein in several disease conditions such as cancer, metabolic disorders, and neurological illnesses. SREBP-1 has two main isoforms: SREBP-1a and SREBP-1c. These isoforms are derived from the same gene but differ in their N-terminal sequences due to alternative promoter usage and splicing [14,15,16,17].

SREBP-1a contains a longer N-terminal sequence with a high percentage of acidic amino acids, making it a potent transcriptional activator. It is involved in both cholesterol and fatty acid synthesis and is expressed at higher levels in tissues with high demands for lipid synthesis, such as during rapid cell division [15,16,17]. SREBP-1c is shorter, lacking 28 amino acids present in SREBP-1a, and contains four unique amino acids. It is a weaker activator of gene expression and is primarily involved in fatty acid synthesis and insulin-induced glucose metabolism. This isoform is predominant in the liver and adipose tissue, where it plays a key role in lipogenesis [15,16,17]. While SREBP-1c is mainly regulated by insulin, SREBP-1a is more responsive to sterol levels [14,15,16,17].

There are also differences in how these isoforms regulate their downstream effectors. SREBP-1a has a stronger ability to activate gene expression due to its interaction with transcriptional coactivators like CBP and P300, whereas SREBP-1c does not interact as efficiently with these coactivators. The expression ratio of SREBP-1a to SREBP-1c varies significantly across different tissues, reflecting their distinct roles in lipid metabolism.

Both isoforms are synthesized as inactive precursors bound to the endoplasmic reticulum membrane. However, for their transcriptional activation, they must bind to SREBP cleavage-activating protein (SCAP) to move their inactive precursors from the endoplasmic reticulum to the Golgi, where they undergo cleavage and subsequent translocation of their NH2-terminal forms to the nucleus. Recent studies have shown that SREBPs are significantly upregulated in human cancers, establishing a mechanistic link between lipid metabolism alterations and malignancies [18]. Inhibition of SCAP or SREBPs, either pharmacologically or genetically, has been found to significantly suppress tumor growth in various cancer models, suggesting that SCAP/SREBPs could be promising metabolic targets for cancer prevention and therapy [19,20].

## 3. Regulation of SREBP1 Activity

As a transcription factor, SREBP1 regulates the expression of genes necessary for lipid synthesis and lipophagy, balancing lipid uptake, storage, and liberation, which is crucial for cell membrane formation and energy storage. These are vital functions in rapidly proliferating cells such as cancer cells. In addition to its role in lipogenesis, SREBP1 contributes to cancer development through its regulatory interactions with glucose metabolism, mitochondrial metabolism, immune cells in the tumor microenvironment, epithelial–mesenchymal transition (EMT), cell cycle, apoptosis, and ferroptosis [18,21]. Liu et al. showed that methylation of arginine in SREBP1a through the activity of arginine methyltransferase 5 (PRMT5) leads to enhanced lipogenesis, increased transcriptional activity, and accelerated cancer cell growth [22]. Conversely, inhibition of SREBP1 activity suppresses lipogenesis and reduces cancer cell proliferation, further highlighting its potential as a therapeutic target [23].

**Figure 1:**
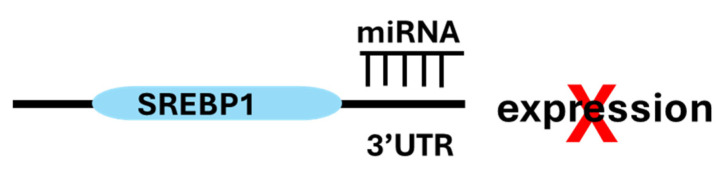
miR-185 and miR-342 target the 3′UTR of SREBP1 mRNA, blocking its expression and thereby inhibiting lipid biosynthesis and tumor cell motility. This effect indicates RNA-based therapeutic targeting of SREBP1 transcription. The light blue capsule represents the mRNA transcribed from the *SREBF1* gene, which can be translated into the SREBP1 protein unless its translation is inhibited.

SREBP1 activity in cancer cells is regulated both transcriptionally and post-transcriptionally by specific miRNAs, such as miR-185 and miR-342, which inhibit cell growth and motility in vitro and in in vivo models of prostate cancer [24]. These miRNAs directly target the 3′UTR of SREBP1 (Figure 1), thereby inhibiting lipid biosynthesis. miRNAs like miR-34a and miR-23a/b modulate upstream regulators of SREBP1 such as mTOR and AKT which have direct roles in cancer development and indirectly modulate lipogenesis by changing the activity of SREBP1, further integrating lipid metabolism with broader tumorigenic pathways [24]. In addition, miR-29 mediates a negative feedback loop regulating the SCAP/SREBP1 signaling pathway and lipogenesis in glioblastoma [19].

**Figure 2:**
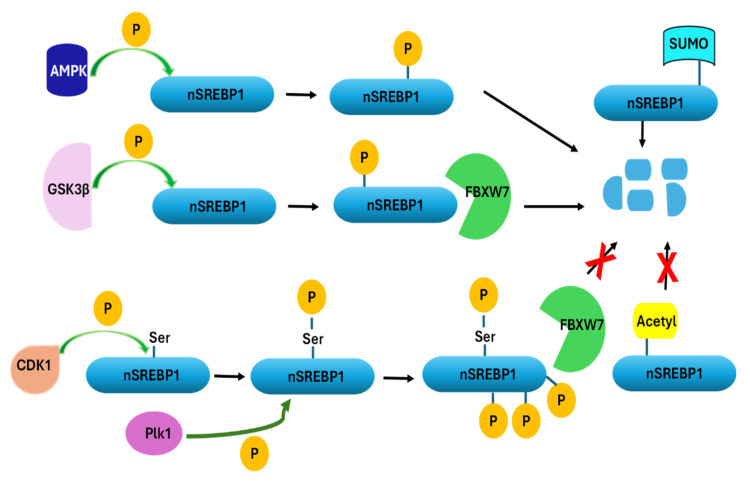
Phosphorylation of nSREBP1 by AMPK or phosphorylation by GSK3β tag nSREBP1 for degradation via FBXW7 leading to reduced levels of SREBP1. Similarly, SUMOylation of nSREBP1 leads to its degradation. Conversely, phosphorylation by mitotic kinases such as CDK1 and Plk1 blocks the interaction between FBXW7 and nSREBP1 and, therefore, stabilizes SREBP1 to support lipogenesis needed for membrane biogenesis and cell division. In addition, acetylation or neddylation of SREBP1 prevent its degradation and maintains its metabolic and transcriptional effects. Solid black arrows indicate the progression of a process. Solid black arrows with red X on them indicate that a process is abolished. Curved colored arrows indicate the addition of phosphate groups to the proteins.

The nuclear forms of SREBPs (nSREBPs) are regulated by post-translational modifications such as phosphorylation, acetylation, and SUMOylation (Figure 2) [23]. Phosphorylation of nSREBP by GSK3β leads to its degradation by FBXW7 (ubiquitin ligase E3 enzyme). However, during mitosis, nSREBP1 is stabilized through phosphorylation of a specific serine residue by the mitotic kinase Cdk1. This results in a close interaction with another mitotic kinase, Plk1, which phosphorylates many residues in the C-terminal domain of SREBP1, blocking interactions with FBXW7 and the degradation of SREBP1 (Figure 2). This stabilization links lipid metabolism with cell proliferation, suggesting that SREBP1 is crucial for cell division [25]. AMP-activated protein kinase (AMPK) activation leads to SREBP phosphorylation and degradation (Figure 2). In contrast, acetylation stabilizes SREBP, and SIRT1 removes acetylation and destabilizes it. Similarly, SUMOylation of nSREBP-1 also results in its degradation (Figure 2) [26]. Another regulatory post-translational modification of SREBP1, which takes place in the cytoplasm, is neddylation, catalyzed by UBC12, which involves attaching a ubiquitin-like molecule Neural Precursor Cell-expressed Developmentally Downregulated 8 (NEDD8) to SREBP1. This process enhances the stability of SREBP1 by reducing its degradation [27]. This indicates that the destabilization of SREBP1 could contribute to reduced cell proliferation and present opportunities for anticancer drug discoveries. Some small molecules and siRNA-based drug candidates have been explored for their efficacy in degrading SREBP1 and reducing cancer cell proliferation [28,29,30,31,32]. However, clinical trials validating their findings are lacking.

SREBP1 is also activated by various signaling pathways. mTORC1 and mTORC2 complexes are involved in regulating SREBP1 activity, with mTORC2 specifically stabilizing SREBP1 to enhance lipogenesis [23,33]. Targeting mTOR pathways, although effective in reducing tumor growth, does not present a suitable strategy for cancer prevention. This is because of the adverse effects of mTOR inhibition, which make it unfit to be used in healthy individuals.

SREBP1 activation is also influenced by mechanical forces from the extracellular matrix (ECM). Actin–myosin contractility and ECM stiffening activate AMPK, which inhibits SREBP1 activation through geranylgeranylated RhoA-dependent pathways [34]. Reduced actomyosin contractility leads to the inhibition of Lipin-1, causing the accumulation of SCAP/SREBP at the Golgi apparatus and subsequent activation of SREBP transcription factors. This process drives lipid synthesis and accumulation, independent of other pathways like YAP/TAZ, mTOR, and AMPK [35]. The regulation of SREBP in response to mechanical cues is also observed in stiffened diseased tissues, contributing to the survival of pluripotent stem cells under certain conditions, such as in the presence of ROCK inhibitors [35]. These data further suggest that despite the central role of SREBP1 in metabolism, it can be targeted independent of broader metabolic effectors such as AMPK and mTOR, promising a more specific interventional approach for cancer prevention with fewer side effects.

**Figure 3:**
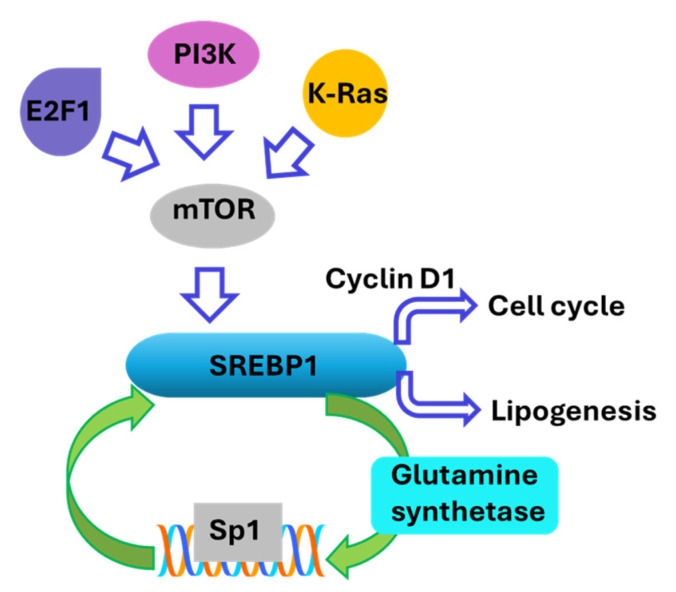
SREBP1 not only spurs lipid synthesis but also forms a feed-forward loop with glutamine metabolism and Sp1, while oncogenes activate it via mTOR signaling. This integration of lipogenic metabolism and the promotion of the cell cycle fuels rapid cell proliferation. The hollow arrows indicate the direction of activation in the designated process, The solid arrows indicate the feed-forward loop.

Another signaling route regulating SREBP1 activity is the endoplasmic reticulum stress and the protein kinase RNA-like endoplasmic reticulum kinase (PERK) pathway. Under endoplasmic reticulum stress, nSREBP1 binds to the promoter of PERK, enhancing endoplasmic reticulum stress responses and autophagy. This interaction amplifies PERK signaling, affecting cell growth and apoptosis [36]. In addition, SREBP1 works synergistically with Sp1 at the low-density lipoprotein receptor (LDLR) promoter. This synergy is crucial for transcriptional regulation in a chromatin context where the accessibility of DNA for gene transcription is impacted by various factors such as histone modifications, nucleosome positioning, and the recruitment of transcriptional activators or repressors to specific regions of chromatin [26]. SREBP1 can also be regulated through feedback loops involving other metabolic pathways, such as glutamine metabolism, which further augments lipid droplet formation in cancer cells [37]. SREBP1 transcriptionally activates glutamine synthetase, resulting in Sp1 O-GlcNAcylation. Subsequently, O-GlcNAc-Sp1 transcriptionally upregulates the expression of SREBP1, resulting in a feedforward loop that increases lipogenesis and lipid droplet formation in liver and breast cancer cells (Figure 3) [37]. These data suggest that breaking the feed-forward loop can disrupt the excess availability of SREBP1 reducing cell proliferation. This could be achieved through reducing the activity of glutamine synthetase that post-translationally activates Sp1, or through reducing certain co-activators such as CBP, disrupting the Sp1 interaction with the promoter region of *SREBF1* that encodes SREBP1.

The role of SREBP1 in cell proliferation is not limited to its regulation of lipogenesis. It activates the expression of cyclin D1, a key regulator of the cell cycle, thereby coordinating cell proliferation with lipid synthesis (Figure 3). This regulation is crucial for the progression through the G1 phase of the cell cycle [38,39]. It supports the metabolic reprogramming necessary for rapid cell division by providing essential lipids for membrane synthesis and energy storage [21,25,40]. It is also known that oncogenes like PI3K, K-Ras, and E2F1 activate SREBP1 through mTORC1 signaling (Figure 3), promoting de novo lipid synthesis and supporting cancer cell growth and proliferation [33,41,42]. These results suggest that reducing the levels or the activity of SREBP1 reduces cell proliferation through its direct effect on cyclin D1, while it also presents SREBP1 activation as a druggable target for reducing the risk of cancers driven by some oncogenes such as PI3K.

**Figure 4:**
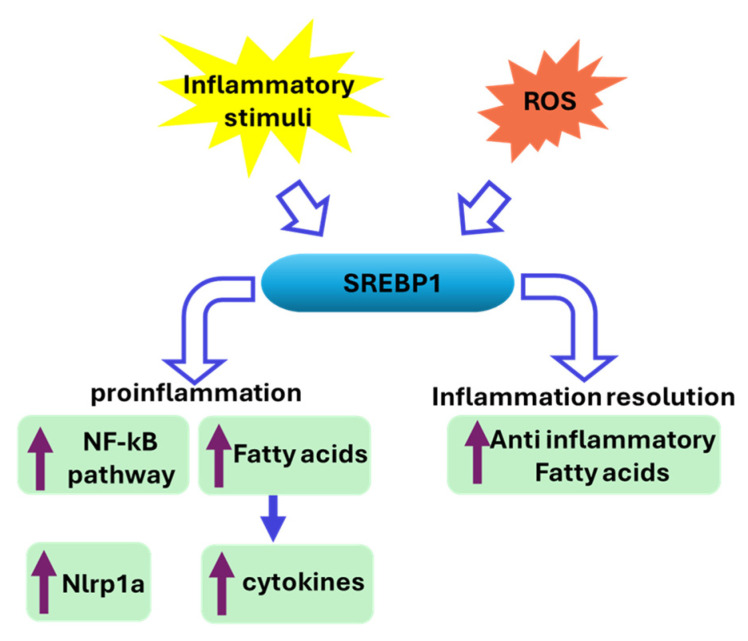
SREBP1 acts as a molecular switch in inflammation: it promotes inflammatory responses in the early phase by activating key pathways and genes but later helps resolve inflammation by reprogramming lipid metabolism and supporting anti-inflammatory processes. These roles are directly tied to its effect on lipogenic metabolism and are reinforced through feedback loops. The hollow arrows indicate the direction of activation in the designated process. The solid purple arrows nside the green rectangles indicate the increase in the designated effector.

In addition to its direct metabolic and proliferative effects, SREBP1 plays a dual role in promoting and resolving inflammation through the polarization of macrophages and lipogenic metabolism (Figure 4) [43]. It facilitates NF-κB activation, promoting the expression of pro-inflammatory genes, upon the inflammatory stimuli [44]. SREBP1 affects the balance of fatty acids and the expression of genes responsible for lipogenesis, thereby impacting cytokine production and inflammation (Figure 4) [45,46]. For example, in keratinocytes, SREBP1 is upregulated in response to obesity and fatty acids, driving the production of inflammatory molecules such as TSLP, which exacerbates skin inflammation in conditions like atopic dermatitis [44]. During the inflammatory response, SREBP1 is activated in M1 (pro-inflammatory) macrophages, particularly through the caspase-11/S1P pathway, which enhances its activation in response to lipopolysaccharide (LPS) treatment and phagocytosis [47]. Additionally, SREBP1a influences the expression of inflammation-related genes, such as the inflammasome sensor Nlrp1a, which is downregulated in SREBP1a-deficient mice, indicating its role in promoting inflammation [48]. This has been observed in dairy cows with fatty liver, where SREBP1c hyperactivates the NF-kB inflammatory pathway by increasing reactive oxygen species (ROS), further increasing hepatic inflammation and injury [49]. The connection of SREBP1 with regulating inflammation is an important mechanism in driving a variety of cancers and presents simultaneous targeting of lipogenesis and inflammation as a strong strategy to retard tumorigenesis.

SREBP1 also contributes to the resolution phase of inflammation by reprogramming fatty acid metabolism in M2 (anti-inflammatory) macrophages. It facilitates the production of anti-inflammatory fatty acids, which help downregulate pro-inflammatory mediators and uncouple NFκB binding from gene activation [43]. This reprogramming is crucial for resolving inflammation and promoting tissue repair, as seen in skeletal muscle regeneration [45,50].

Collectively, these studies suggest that SREBP1 sits at the intersection of at least two hallmarks of cancer, reprogramming metabolism and inflammation. These features are common to many cancers, including breast cancer [51], where the fatty composition of the microenvironment, which is susceptible to inflammation, is associated with malignant transformation.

## 4. The Implication of SREBP1 in Various Cancers

Suppression of SREBP1 or its degradation has been shown to inhibit lipogenesis, fatty acid oxidation, and tumor growth in vitro and in vivo models of colon cancer [52]. The effect of the EGFR-tyrosine kinase inhibitor, gefitinib, at suboptimal doses was reported to be potentiated in the presence of SREBP1 inhibitors such as betulin and fatostatin in models of non-small cell lung cancer [53]. Similarly, SREBP1 was reported to enhance the resistance of lung squamous cell carcinoma cells to antitumor drugs such as gefitinib, anlotinib, and osimertinib to name a few. A small molecule inhibitor of SREBP1, the natural product monomer compound 3-(5-isopropyl-4-(4-methylpyridin-3-yl) thiazol-2-yl) benzamide, exhibited inhibition of SREBP1 activity, reversal of the Warburg effect, and suppression of EMT in these cells, while also increasing sensitivity to antitumor drugs [30,54]. In head and neck cancers, upregulation of *SREBF1*, the gene encoding SREBP1, promotes proliferation and migration, and it inhibits apoptosis by upregulating the expression of steroidogenic acute regulatory protein-related lipid transfer 4 (STARD4) and modulating the level of immune cell infiltration [55]. It has also been shown that silencing SREBP1 inhibits the proliferation and motility of human esophageal squamous carcinoma in vitro and in vivo [56]. This is via the downregulation of SCD1, phosphorylated GSK3β, and nuclear β-catenin in the wnt/β-catenin signaling pathway [56]. Other studies have suggested that a small molecule inhibitor of SREBP1, SI-1, could be a promising treatment for advanced hepatocellular carcinoma and that betulin enhances the antitumor effect of Sorafenib on hepatocellular carcinoma via modulating metabolism [57,58]. In addition, inhibition of Sp1, which activates SREBP1 (Figure 4), led to reduced proliferation in vitro and regressed tumor growth in vivo in models of renal cell carcinoma [59]. SREBP1 overexpression was demonstrated to promote progesterone resistance in models of endometrial cancer and increase their proliferation through activation of the NF-kB pathway and inhibiting apoptosis [60]. Inhibition of SREBP1 with fatostatin restored sensitivity of endometrial cancer to progesterone in vitro and in vivo [60]. These data are a confirmation that activation of SREBP1 promotes inflammatory mediators to support tumor growth in a variety of malignancies.

In nutrient-deprived conditions, tumor cells not only increase their de novo lipogenesis to support proliferation but SREBP1 also upregulates the expression of the lysosome cholesterol transporter, NPC2, and genes involved in autophagy [21]. This results in the release of cholesterol and fatty acids to maintain tumor growth [21]. Similarly, oncogenic mutations activating the PI3K–AKT–mTORC1 pathway (e.g., PIK3CA mutations, PTEN deletions) confer ferroptosis resistance in cancer cells by upregulating SREBP1 and its downstream effector, SCD1 [61]. This pathway enhances monounsaturated fatty acid (MUFA) production, which suppresses lipid peroxidation, thereby inhibiting ferroptosis. This has been confirmed by using fatostatin to inhibit SREBP1, which sensitizes cancer cells with PI3K-AKT-mTOR mutations to ferroptosis inducers. Combination therapy with mTORC1 inhibitors and ferroptosis inducers results in synergistic tumor regression in xenograft models of breast and prostate cancers [61]. A similar mechanism was revealed in non-small cell lung cancer in which the G protein-coupled estrogen receptor activated the SREBP1/SCD axis to suppress ferroptosis [62]. These examples reveal how genetic factors of cancer development use SREBP1 as a robust tool to exert tumorigenesis and suggest disruption of SREBP1 activity as a way to block oncogene-driven cancers.

## 5. SREBP1 and Breast Cancer

In normal breast tissue, SREBP1 is involved in maintaining lipid homeostasis and supporting normal cellular functions and milk production [40,63]. From pregnancy to lactation, the expression of SREBP1 significantly increases [64]. Overexpression of SREBP1 in mammary cells leads to increased expression of genes involved in fatty acid activation, transport, desaturation, and de novo synthesis, resulting in higher triacylglycerol content necessary for milk production [64]. Decreased SREBP1 activity, such as during diet-induced milk fat depression, leads to reduced expression of lipogenic enzymes and lower milk fat synthesis, further highlighting its essential regulatory role [65]. The activity of SREBP1 is modulated by other regulatory proteins and factors, such as INSIG1 and S14, which also influence the overall lipid synthesis process in the mammary gland [64,65]. SREBP1’s role in normal breast physiology is distinct from its involvement in breast cancer, where its dysregulation can contribute to tumor growth and metastasis, as follows:

In breast cancer, SREBP1 is required for de novo lipogenesis that is sometimes consequent to oncogene activation and growth-factor independent proliferation of transformed breast cells [33]. In ER+ breast cancer cells, 17β-estradiol was shown to induce a significant increase in SREBP1 activity and its downstream effector SCD1; the effect being absent in non-malignant MCF-10A cells [66]. It was further revealed that 17β-estradiol not only stimulates the expression of SREBP1 but also promotes its maturation into the active nuclear form, connecting estrogen signaling with lipid metabolic reprogramming in ER+ breast cancer [66]. It is important to note that estrogen represses SCD-1 in metabolic tissues but activates it in ER+ breast cancer cells, and that activation of SCD-1 via SREBP1 is central to estrogen-induced proliferation, thereby positioning the SREBP1–SCD1 axis as a therapeutic target for ER+ breast cancer [67,68]. Similarly, high expression levels of *SREBF1* (gene encoding SREBP1) are correlated with poor recurrence-free survival (RFS) and distant metastasis-free survival (DMFS) in breast cancer patients [69]. SREBP1 levels are highly correlated with breast tumor differentiation, tumor-node metastasis (TNM) stage, and lymph node metastasis. In addition, it predicts poor prognosis and is an independent factor of 5-year overall and disease-specific survival in breast cancer [70].

A study in MDA-MB-231 (ER−), MCF-7 (ER+) cancer cells, and non-transformed MCF-10A cells as well as xenograft models showed that polyphenols vitexin and syringic acid reduced proliferation and mammary tumor growth by decreasing the protein expression of SREBP1. Interestingly, further studies showed that while suppression of SREBP1 did not reduce the lipid contents of the cells, it specifically reduced the ratio of monounsaturated fatty acids/saturated fatty acids (MUFA/SFA) by reducing the level of SCD1, which affects the membrane fluidity and stagnates proliferation [71]. This is in line with the earlier studies on microenvironmental factors associated with breast tumorigenesis, which indicated SCD1 has a definitive role in membrane fluidity and enhanced migration of breast cancer cells [68]. Interestingly, although SCD1 was shown to be essential for cell migration, there was a divergence in mechanism between poorly invasive MCF-7 and the highly aggressive MDA-MB-231 cells, when they were co-cultured with cancer-associated fibroblasts. In MCF-7 cells, SREBP1 protein expression and its DNA-binding activity were significantly increased, while in MDA-MB-231 cells, despite a reduction in SREBP1 protein expression, DNA binding and transcriptional activity were enhanced, promoting the migration of both cell lines [68]. These studies provide evidence that targeting downstream effectors of SREBP1 such as SCD1 as a druggable target can successfully reduce the oncogenic activities of SREBP1 without disrupting its other beneficial roles such as resolving inflammatory/immune cell responses.

Increased nutrient sensing through elevated post-translational O-GlcNAcylation is associated with oncogenesis, and the enzyme O-GlcNAc transferase (OGT), which is responsible for this modification, is overexpressed in cancers. OGT has been shown to control the transcription and post-translational modification of SREBP1 and its downstream effectors [72]. A reduction in OGT in breast cancer cells led to decreased SREBP1 activity, lower lipogenesis, and impaired tumor growth in vitro and in vivo. In addition, OGT has been shown to indirectly stabilize SREBP1 by modulating AMPK activity. A reduction in OGT levels activates AMPK, phosphorylating SREBP1 at Ser372, which prevents its nuclear translocation and activation [73]. This study presents additional evidence that targeting the stabilization of SREBP1 is a promising approach to curbing tumor growth by minimizing the nutrient-sensing capacity of SREBP1. These strategies can be applied to the prevention of breast cancer if they are established in pre-cancer models and verified in non-invasive breast tumors such as in situ disease.

### 5.1. SREBP1 and Ductal Carcinoma In Situ of the Breast

SREBP1 is significantly upregulated in cancer stem-like cells (CSCs) of breast ductal carcinoma in situ (DCIS) and is associated with increased lipogenesis [74]. The elevated expression of SREBP1 and its downstream targets, such as ACLY, ACC-1, and FASN, is observed in both clinical specimens and cell lines of DCIS, suggesting a critical role in the early stages of breast tumorigenesis [74]. Cancer stem-like cells isolated from DCIS exhibit higher levels of lipogenic gene expression compared to non-tumorigenic cells. This enhanced lipogenesis is linked to increased tumor-initiating ability and survival of CSCs, indicating that lipogenesis is a prerequisite for DCIS formation. The overexpression of SREBP1 in non-tumorigenic cells can induce similar lipogenic activity, promoting cell growth and mammosphere formation, which are indicative of stemness and tumorigenic potential [74]. Similarly, oleic acid (OA) has been shown to increase proliferation, migration, and lipogenic protein expression, such as SREBP1 expression, in MCF10DCIS.COM cells, a model for DCIS, particularly in ALDH^high^ subpopulations, which are more prone to cancer progression [75].

Tamoxifen, already established as a breast cancer prevention drug, increases the risk of non-alcoholic fatty liver disease (NAFLD) by promoting triglyceride (TG) synthesis in the liver. This is consequent to the upregulation of SREBP-1c expression through an ER/LXR-dependent pathway, resulting in excessive TG accumulation in hepatocytes and liver steatosis [76]. Tamoxifen facilitates this process by enhancing the recruitment of the co-activator p300 and dissociation of the co-repressor NCOR within the ER (estrogen receptor)/LXR/RXR complex. This transcriptional shift boosts SREBP-1c activity, promoting lipid dysregulation. Vitamin D3 (VD3) reverses the tamoxifen-induced transcriptional activation of SREBP-1c by modifying cofactor dynamics in the ER/LXR complex: it promotes NCOR recruitment and induces p300 dissociation, thereby repressing SREBP-1c expression and mitigating hepatic TG accumulation. Notably, this protective effect does not involve direct interaction with the Vitamin D Receptor (VDR), indicating an alternative VD3 mechanism via ER/LXR complexes. In a mouse model, tamoxifen increased TG levels and lipid deposition in the liver, evidenced by histological (H&E and Oil Red O staining) and biochemical analyses. Supplementation with VD significantly alleviated tamoxifen-induced lipid accumulation by reducing hepatic TG content and downregulating SREBP-1c expression. These findings establish VD3 as a potential adjunctive treatment to counteract tamoxifen-associated NAFLD, offering a novel therapeutic strategy based on SREBP1 activity to prevent lipid metabolism disorders in breast cancer patients undergoing tamoxifen therapy [76]. In addition, inhibition of the SREBP1-dependent enzyme ACLY has been reported to enhance the efficacy of tamoxifen in breast cancer cells; ACLY additionally is a potential biomarker for predicting breast cancer recurrence [77,78].

### 5.2. SREBP1 and Invasive Progression of Breast Cancer

In addition to the formation of DCIS, SREBP1 is implicated in promoting EMT in the breast by forming a complex with Snail and HDAC1/2, which leads to the repression of E-cadherin-promoting EMT, migration, metastasis, and unfavorable clinical outcomes in breast cancer [32]. Functional studies have shown the binding of miR-215-5p to the 3′UTR of SREBP1 mRNA, which leads to SREBP1 downregulation. N-acyl dopamines, including N-arachidonoyl dopamine, have been shown to reduce breast cancer cell migration and colony formation, reduce EMT markers, and enhance epithelial marker expression, by suppressing SREBP1. Their mechanism of action was reported to be through the inhibition of ERK1/2 phosphorylation, without affecting PI3K–AKT signaling, linking ERK inhibition to SREBP1 suppression [79]. Moreover, SREBP1 overexpression leads to breast cancer cell migration and invasion; suppression of SREBP1 significantly inhibits these processes [68,70]. Similarly, increased FASN in conjunction with enhanced mesenchymal–epithelial transcription factor (MET) in triple-negative breast cancer were positively correlated with lymph node metastasis, stage, recurrence, metastasis, and survival [80].

Breast cancer cells and their xenografts have shown significant sensitivity to leucine deprivation, while normal cells such as primary hepatocytes and brown adipose tissue were not affected, suggesting a therapeutic selectivity [81]. The study showed that leucine deprivation reduced FASN expression by suppressing SREBP1c. They demonstrated that GCN2 (General Control Nonderoncol lettepressible 2) upstream of SREBP1 was activated, which led to the reduced expression of SREBP1c. Leucine deprivation induced apoptosis in breast cancer cells as evident by Annexin-V staining and elevated cleaved caspase-3 and -8 in MDA-MB-231 and cleaved caspase-9 in MCF-7 cells. Apoptosis was also observed in the xenograft tumors in the leucine-deprived diet group, evident by TUNEL-positive staining. These effects were reversed by the supplementation of palmitic acid (a product of FASN activity) and highlighted the importance of targeting the GCN2-SREBP1c/FASN axis to intercept breast carcinogenesis [81].

Lipid metabolism reprogramming, facilitated by SREBP1 expression, enables triple-negative breast cancer (TNBC) cells to adapt and survive under hypoxia and nutrient-deprived conditions. It is known that hypoxia triggers autophagy in TNBC cells and works in synergy with SREBP1-mediated lipid reprogramming. Autophagy helps recycle intracellular components, including fatty acids, to sustain energy production and cell survival. These effects have been shown by fatostatin (a specific inhibitor of SREBP1), which reduces TNBC cell viability under hypoxic conditions. It was suggested that targeting SREBP1 combined with inhibitors of fatty acid oxidation and autophagy could present an effective strategy for TNBC treatment [69]. However, the precise mechanism of SREBP1 activity under hypoxic conditions still needs to be elucidated. In addition, the application of this strategy in preventing TNBC formation or progression need to be comprehensively studied.

One of the SREBP1-dependent adaptive strategies for TNBC cells happens at the chromatin level. FLAD1, located at chromosome region 1q21.3, is frequently amplified in TNBC, contributing to poor prognosis by driving tumor progression. Mechanistically, FLAD1 enzymatic activity facilitates the conversion of FMN to FAD, which activates LSD1 (a histone demethylase). LSD1 demethylates H3K9me2 at the promoter of SREBP1, leading to its upregulation at the transcriptional level [82]. The LSD1–SREBP1 axis promotes the expression of lipogenic enzymes (e.g., FASN, ACC1, SCD), enhancing TNBC growth and lipid biosynthesis. Knockdown of FLAD1 or inhibition of LSD1 reduces lipid content and suppresses TNBC malignancy. Targeting the FLAD1–LSD1–SREBP1 axis with inhibitors like GSK-LSD1 (LSD1 inhibitor) or fatostatin (SREBP1 inhibitor) significantly reduces TNBC growth in vitro and in vivo. Combination therapies involving LSD1 inhibitors and standard chemotherapeutic agents (e.g., doxorubicin, sacituzumab govitecan) show synergistic effects in reducing tumor growth.

In endocrine-resistant breast cancer models such as aromatase inhibitor-resistant MCF-7 cells, SREBP1 not only activates lipogenesis and the formation of hydroxy cholesterol, re-activating ERα and enhancing cancer cell survival, but it also drives the upregulation of Keratin-80 (*KRT80*) by binding to the enhancer region of the gene and activating its transcription [83]. The SREBP1-mediated upregulation of *KRT80* results in significant cytoskeletal rearrangements, such as increased cellular stiffness and the formation of actin-rich lamellipodial structures. These results are in line with the findings of other studies which showed that SREBP1 is activated through mechanical cues [34,35]. These changes assist with increasing the invasion of cancer cells. As a result, low-risk breast cancer evolves into a high-risk disease. These data are also supported by clinical evidence demonstrating the link between SREBP1-mediated KRT80 upregulation and enhanced tumor stiffness, poor prognosis, and earlier relapse in patients receiving endocrine therapies [83]. These observations may suggest that reducing the activity of SREBP1 in conjunction with the standard of care therapy leads to more favorable outcomes in patients with endocrine-resistant breast cancers.

Neddylation, the stabilizing post-translational modification of SREBP1 through the catalytic activity of UBC12, has been shown to have a role in promoting breast cancer, increasing EMT, invasion, and metastasis, primarily by fueling lipogenesis [84]. Similarly, in clinical samples, particularly in metastatic cases, the levels of SREBP1 and UBC12 are elevated in the breast cancer tissue [84]. These observations once again affirm the key role of neddylation in promoting SREBP1 effects and the importance of targeting SREBP1 stability for cancer prevention and interception.

A novel regulator of SREBPs is the Death domain-associated protein X (DAXX) with roles in transcription regulation and chromatin remodeling. DAXX has been reported to interact directly with both the full-length and mature forms of SREBP1/2 [85]. A comprehensive study revealed that DAXX expression levels positively correlate with de novo lipogenesis in TNBC cells. These findings were validated by lipidomic profiling, and they demonstrated DAXX binding to SRE as an activator at the promoter of SREBP-mediated lipogenic genes. They demonstrated when the functional DAXX’s SUMO-interacting motifs (SIMs) were absent, its binding to SREBPs, chromatin recruitment, and transcriptional activation of lipogenic genes were disrupted. The study also showed that in xenograft models targeting DAXX–SREBP interaction with a synthetic peptide (SIM2) that blocks DAXX–SUMO interaction inhibited lipogenesis and tumor growth, presenting the DAXX–SUMO interface as a therapeutic strategy to curb SREBP-dependent lipogenesis and tumor growth [85].

NR4A1 is a regulator of lipid metabolism in non-cancer tissues such as the liver and can reduce SREBP1 expression and activity in cancer cells. This regulation is through suppressing the transcription and activity of SREBP1 target genes, such as FASN and SCD1, which suggests that NR4A1 agonists may potentially be useful in managing SREBP1-driven cancers [86].

It is important to note that macrophages, which depend on lipid metabolism, contribute to an immune-suppressive microenvironment, and inhibition of SREBP1 reverses this phenotype [87]. It has been shown that in tumor-bearing K14-Cre; *Brca1*^f/f^; *p53*^f/f^ mice that represent *BRCA1*-associated TNBC, SREBP1 contributes to the PARP inhibitor resistance and that the inhibition of SREBP1 can reverse the metabolic state of macrophages and the resistance [88]. This observation further confirms the multifaceted functions of SREBP1 and that its activity needs to be well-defined and fine-tuned to block tumor growth. In addition, its modulation of immune response can be an added benefit when SREBP1 inhibitors are combined with the standard of care treatments in TNBC disease.

### 5.3. SREBP1 and Oncogene-Driven Breast Cancer

It has been shown that SREBP1 activity is required for the palmitoylation of Ras in the progression and metastasis of Ras-dependent breast cancers, and that suppression of transcriptional activity of SREBP1 through the interaction of CLDN6 with MAGI2 and KLF5 reduces palmitic acid biosynthesis and palmitoylation of Ras [89]. This results in reduced ERK activity and cyclin D1 expression, leading to reduced tumor growth and metastasis.

The tumor suppressor RBP7 (Retinol-Binding Protein 7) is linked to lipid metabolism and the PI3K–AKT pathway. It was shown that RBP7 is significantly downregulated in hormone receptor-positive (HR+) breast cancer tissues compared to normal tissues, and its low expression correlates with poor overall survival (OS) and higher tumor T-stage and Ki67 score [90]. Mechanistically, RBP7 induced G0/G1 cell cycle arrest and reduced fatty acid levels in HR+ breast cancer cells and xenograft models. This was performed through the inhibition of the AKT–SREBP1 signaling pathway, reducing the expression of AKT, phosphorylated AKT (p-AKT), and SREBP1. This study highlighted RBP7 as a novel prognostic biomarker and is another piece of evidence that the SREBP1-dependent lipogenesis is a promising target to disrupt tumorigenesis and tumor progression [90].

One of the nuclear proteins that is upregulated in human breast cancer tissues compared to the adjacent normal tissues is p54^nrb^, which is involved in RNA editing, DNA repair, and gene transcription. Its role in promoting breast tumor growth is linked to its direct interaction with SREBP1a, which stabilizes the active form in the nucleus, increasing lipogenesis and proliferation [85]. Increased SREBP1 expression activates lipid biosynthesis genes, supporting the increased demand for hyperproliferating cells. It interacts with proteins such as p54nrb/NONO and the oncoprotein HBXIP (hepatitis B X-interacting protein) leading to enhanced lipid production, which is essential for the cellular energetics and membrane biosynthesis of the proliferating cancer cells [91,92].

In various non-malignant and malignant breast cell lines the oncogenic effects of PI3K and K-Ras are mediated through enhanced mTORC1 and SREBP activity [33]. In breast cancer patients, increased expression of the oncoprotein HBXIP, which is associated with decreased overall and progression-free survival, exerts its effects through the upregulation of SREBP-1c and activating LXR (liver X receptor) in a ligand-independent manner. This leads to the enhanced expression of FASN, and this effect is reinforced through a positive feedback loop between SREBP-1c and HBXIP [92].

Another oncoprotein, Myc, binds to the promoter region of the SREBP1 gene and activates its transcription. It has been shown that circMyc, a circular RNA generated from the Myc gene in TNBC tissues and cell lines, correlates with larger tumor size, lymph node metastasis, advanced TNM stage, and poorer overall survival. circMyc amplifies de novo fatty acid synthesis by regulating SREBP1 at both transcriptional and post-transcriptional levels [93]. circMyc binds to HuR (an RNA-binding protein) in the cytoplasm, enhancing HuR’s stabilization of SREBP1 mRNA and reducing mRNA decay. In addition, circMyc interacts with Myc protein in the nucleus, facilitating Myc binding to the SREBP1 promoter, which enhances SREBP1 transcriptional activity [93].

In a study on TNBC, it was observed that dual inhibition of CDK4/6 and CDK7 suppressed tumor growth by reducing SREBP1 without having a significant effect on SREBP2; nevertheless, downstream cholesterol biosynthesis was lowered [94]. The study showed that FOXM1 (Forkhead Box M1) levels and its occupancy on the *SREBF1* promoter were reduced, which led to the reduction in SREBP1 levels. In addition, the dual inhibitors disrupted the interaction of SREBP1 with the histone acetyltransferase P300, leading to reduced transcriptional activation of cholesterol biosynthesis genes. In clinical data sets patients with higher expressions of *FOXM1*/*SREBF1* or SREBP1-related genes exhibit worse outcomes in TNBC, highlighting the prognostic significance of this pathway [94].

A regulatory step in lipid homeostasis and SREBPs’ activity is the ABCA family of lipid transporters. It has been shown that ABCA9 localizes to endoplasmic reticulum and accumulates cholesterol in this organelle [95]. Its overexpression inhibited the nuclear translocation of SREBP2, reducing the expression of cholesterol biosynthesis genes. ABCA9 expression was downregulated in breast cancer cells and patient samples, and its restoration reduced proliferation and colony formation, and enhanced apoptosis. These investigators also found that ABCA9 is under the control of FOXO1 (tumor suppressor), which is downregulated in breast cancer, due to the activation of the PI3K–AKT pathway, which phosphorylates FOXO1. Inhibition of PI3K led to the restoration of FOXO1 activity and increased ABCA9 expression. The study suggests the PI3K–FOXO1–ABCA9 axis as a potential therapeutic target for breast cancer treatment [95].

### 5.4. SREBP1 and Obesity-Driven Breast Cancer

It has been shown in vitro and in vivo that the role of adiponectin in blocking breast tumorigenesis is associated with the suppression of SREBP1-dependent lipogenesis and activation of lipophagy-mediated lipolysis and fatty acid oxidation [96]. Conversely, tumorigenic effects of leptin in the breast are through the upregulation of SREBP1 in its precursor form and cleaved forms via the activation of PI3K signaling, phosphorylation of AKT, and autophagy, with the latter being independent of PI3K signaling [97].

A single-cell RNA sequencing study on breast tumors from obese and non-obese women showed that NR1H3 is upregulated in a subset of obesity-specific macrophages. This transcription factor binds to SREBP1 promoting its expression and the subsequent increase in FABP4 (fatty acid binding protein 4), leading to breast cancer cell proliferation [98].

Visfatin is an adipokine elevated in obesity and breast cancer patients, promoting cancer progression. It has been demonstrated that visfatin stimulates SREBP1 expression and activity in MCF-7 breast cancer cells [99]. This study revealed that visfatin binds to EGFR and triggers PI3K activation, leading to AKT phosphorylation at Ser473. Activated AKT phosphorylates GSK3β at Ser9, inactivating it. This removes the inhibitory effect of GSK3β on SREBP1 (Figure 2), allowing SREBP1 to mature, translocate to the nucleus, and activate target gene transcription. The study employed chemical (fatostatin) and genetic (siRNA) inhibitors of SREBP1 to demonstrate the importance of lipid synthesis in visfatin-stimulated MCF-7 cells [99]. Their findings suggest that targeting the EGFR–PI3K–AKT–GSK3β–SREBP1 axis or inhibiting SREBP1 directly could serve as potential therapeutic strategies for breast cancer, especially in obesity-associated cases where visfatin levels are elevated. These studies suggest that SREBP1 is directly involved in the development of obesity-driven breast cancer, and its suppression or destabilization could reduce the risk of breast cancer in women with obesity.

### 5.5. SREBP1 and Metastasis of Breast Cancer

High expression levels of *SREBF1* (gene encoding SREBP1) are correlated with poor recurrence-free survival (RFS) and distant metastasis-free survival (DMFS) in breast cancer patients. These findings underline the prognostic significance of SREBP1 and its potential as a biomarker for aggressive breast cancer [82]. A study showed that the overexpression of *ZSWIM3* (zinc-finger SWIM domain-containing protein 3) in MCF-7 cells promotes breast cancer progression and metastasis by enhancing the levels of *SREBF1*, *SREBF2*, and the levels of lipids, the effect that was reversed when *ZSWIM3* was knocked out [100]. A breakthrough study further confirmed the reliance of metastasis on lipogenic metabolism when they demonstrated that lipidomic profiling using Raman Spectroscopy can stratify organ-specific metastasis and identify lipid metabolism-targeting therapies [101].

Enhanced SREBP1 and lipogenic metabolism make breast cancer cells brain trophic [101]. Brain metastases are known to exhibit distinct metabolic adaptations to the glucose-deprived, cholesterol-rich microenvironment of the CNS; breast–brain metastasis relies heavily on lipid biosynthesis pathways regulated by SREBP1 [101]. A single-cell transcriptomic study found that *RARRES2* (Retinoic Acid Receptor Responder 2) is the most downregulated gene in brain-metastatic TNBC tumors compared to primary breast tumors [102]. The loss of *RARRES2* activates the PTEN–mTOR–SREBP1 signaling axis, promoting lipid metabolic reprogramming required for brain metastasis formation. Overexpression of *RARRES2* inhibits cell proliferation, invasion, and brain metastases in both intracranial and intracardiac in vivo models [102]. These findings suggest targeting lipid metabolism or components of the PTEN–mTOR–SREBP1 pathway, early on might serve as preventive or therapeutic strategies for TNBC brain metastasis. Additionally, combining standard-of-care treatments with modulators of SREBP1-dependent metabolism may enable dose de-escalation and intermittent dosing strategies, potentially reducing toxicity while maintaining therapeutic efficacy.

## 6. SREBP1 and Lobular Breast Cancer

Lobular carcinoma in situ (LCIS) is relatively uncommon but is a clinically significant breast lesion, often detected incidentally through biopsies performed for various reasons. Its existence increases the risk of invasive breast cancer by 9–10 folds compared to the general population and, therefore, presents opportunities for prevention [103,104]. Analysis of invasive lobular carcinoma (ILC) of the breast from a letrozole treatment trial revealed that tumors from non-responders exhibited increased SREBP1 expression compared to responders, connecting SREBP1 to endocrine resistance development [105]. These observations were also valid in long-term estrogen-deprived cells, which showed overexpression of SREBP1 and its downstream targets that support survival under estrogen deprivation. These observations position SREBP1 as a promising therapeutic target for endocrine-resistant ILC [105]. In addition, it could provide a basis for preventive studies focused on women who are diagnosed with LCIS, in order to prevent development of the invasive disease.

### 6.1. Differential Role of SREBP1 in Breast Cancer Subtypes

SREBP1 is often upregulated in TNBC and HER2+ breast cancers, which have a greater reliance on de novo lipogenesis, compared to luminal breast cancers [106]. This dependence is more pronounced under hypoxic and nutrient-deprived conditions, and when these tumors metastasize [32,107,108,109].

**Figure 5:**
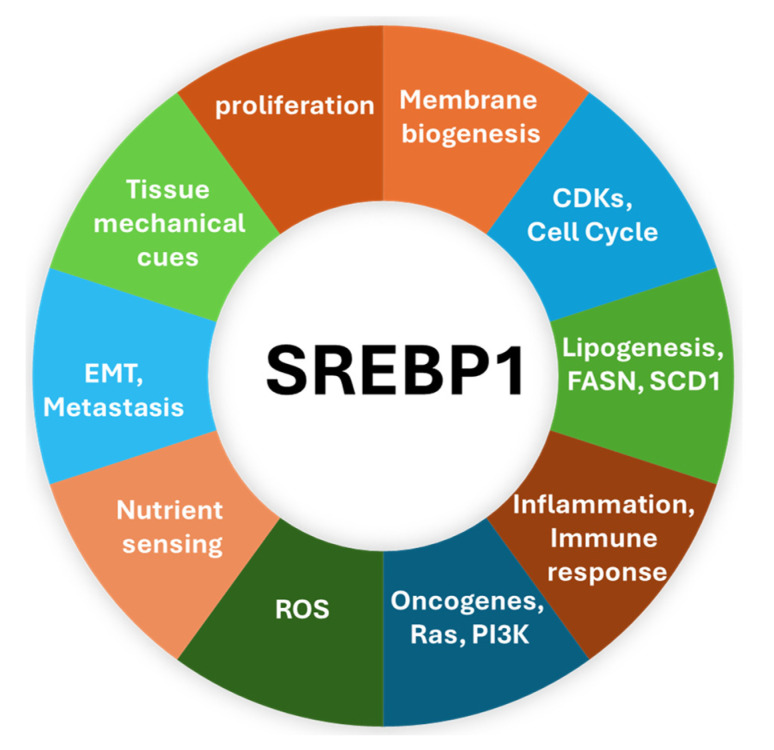
SREBP1 contributes to the development of breast cancer through various mechanisms. In addition to its role in lipogenic metabolism through the activity of fatty acids and cholesterol biosynthesis, it is closely tied to various factors at the cell and tissue microenvironment levels regulating malignant transformation and cancer progression, as depicted in this figure. Targeting these pathways interrupts tumor initiation, progression, and metastasis.

As discussed above, there is strong evidence of a direct correlation between increased SREBP1 activity and the progression, survival, and poor prognosis of TNBC. SREBP1 expression is upregulated in TNBC through several pathways, including the FLAD1/LSD1 axis and the mTORC1 signaling pathway, both of which enhance lipid biosynthesis and support tumor progression [82]. In hypoxic conditions, TNBC cells show increased SREBP1 activation, which boosts lipogenesis, autophagy, and fatty acid oxidation, all contributing to cell survival and proliferation [69]. As discussed earlier and shown in Figure 5, SREBP1 promotes EMT and metastasis, by repressing E-cadherin and forming a co-repressor complex with Snail and HDAC1/2 in the TNBC setting [32]. Consistent with these reports, inhibition of SREBP1, either directly or through upstream regulators, has been shown to reduce TNBC cell viability, impair tumor growth, and enhance the effectiveness of existing chemotherapies [94]. SREBP1 drives key metabolic and survival pathways in TNBC, is linked to poor prognosis, and represents a promising target for new therapies.

Although the direct effect of HER2/neu activation on SREBP1 activity has not been well studied, there is a strong correlation between the activity of HER2/neu and the increased activity of the mTOR pathways, upstream of SREBP1. HER2/neu, often in partnership with ErbB3, activates the PI3K–Akt pathway, which in turn stimulates mTOR activity [110,111]. This includes upregulation of mTORC1 pathway components such as p85/PI3K and p70S6 kinase, and downregulation of inhibitors like eIF-4E-BP1, all of which enhance mTORC1 signaling. Tumors with HER2/neu activation show increased phosphorylation of ribosomal protein S6, a marker of mTORC1 activity, indicating hyperactivation of this pathway. Inhibition of mTOR (including mTORC1) with drugs like rapamycin in vitro and in vivo leads to tumor growth arrest and regression in HER2/neu-driven cancer models, further supporting the role of mTORC1 downstream of HER2/neu and upstream of SREBP1. In addition, the loss of tumor suppressors like LKB1 in HER2/neu models further increases mTORC1 activity, accelerating tumor development, which highlights the central role of mTORC1 in HER2/neu-mediated oncogenesis [112]. Consistent with these observations, hyperactivation of mTORC1 is associated with resistance to HER2-targeted therapies, and combining mTORC1 inhibitors with HER2 inhibitors can restore drug sensitivity in resistant cancers [113]. These findings support the rationale for targeting mTORC1 in HER2/neu-positive cancers to improve therapeutic outcomes; however, direct downregulation of SREBP1-dependent pathways to block the effects of HER2/neu activation or to overcome therapy resistance needs further investigation.

Luminal (hormone-responsive) breast cancers are less lipogenic and more reliant on oxidative metabolism and estrogen-regulated genes. The direct interplay between SREBP1 activity and the pathogenesis of luminal breast cancer is not well-established; however, strong evidence exists supporting a cross-talk between estrogen signaling and lipid metabolism in breast cancer. For example, estrogen signaling shifts breast cancer cell metabolism toward increased glycolysis and lipid synthesis, especially when combined with progestins [114,115]. This metabolic adaptation supports tumor progression and survival under stress. More directly, it has been shown that ERα directly regulates genes involved in choline and phospholipid metabolism, such as *CHPT1*, leading to increased phosphatidylcholine synthesis and supporting cancer cell proliferation and metastasis [116]. In addition, estrogens, through both classical ERs and the G protein-coupled estrogen receptor (GPER), upregulate FASN, important for cancer cell growth and survival [117]. These data present opportunities to disrupt hormone-driven metabolic pathways, such as FASN or mTOR to enhance the effectiveness of endocrine therapies in ER+ breast cancer [118,119]. Another example of such correlation is that estrogen induces the expression of *SLC22A5*, a gene critical for carnitine homeostasis and lipid metabolism. This effect is mediated through an estrogen-responsive element and its disruption affects lipid droplet accumulation and cell proliferation in breast cancer cells [120].

The direct role of SREBP1 in luminal breast cancers is more pronounced when endocrine resistance or PI3K mutations exist. As discussed above, in ILC, which is resistant to endocrine therapies, SREBP1 is upregulated [105]. In addition, the overexpression of SREBP1 is associated with aggressive tumor behavior and poor prognosis [18,70]. Targeting SREBP1 or its downstream lipid metabolic pathways may offer new strategies to overcome endocrine resistance and limit tumor progression in ER+ breast cancers. These studies highlight the importance of targeting both hormonal and metabolic processes in luminal breast cancer treatment.

Overall, TNBC presents the most promising context for therapeutic targeting of SREBP1, while in HER2+ and ER+ settings, combination therapies may assist with overcoming drug resistance and improve outcomes. SREBP1 is a promising target in breast cancer due to its role in tumor progression and therapy resistance. Although direct synergism between SREBP1 inhibitors and chemotherapy or immunotherapy has not been clinically demonstrated, targeting SREBP1 may enhance the effectiveness of these treatments by altering tumor metabolism and immune responses. Further research is needed to validate these combinations in clinical settings.

### 6.2. SREBP1 Inhibitors for Breast Cancer Prevention and Interception

The activity of SREBP1 can be reduced through several targetable pathways, such as by inhibiting its upstream effectors mTOR, PI3K–AKT, and EGFR. However, these targets have broad metabolic consequences, making them not suitable for the cancer prevention setting, although their application in the therapy arena is ongoing. Other targetable routes for reducing the effects of SREBP1 are the pathways associated with its proteolytic activation, its stability, and its trafficking from the endoplasmic reticulum to the Golgi and from the cytoplasm to the nucleus (Table 1). These routes that are specific to SREBP1 would make more plausible targets for cancer prevention interventions. Several small molecules, miRNAs, and proteins have been studied as inhibitors of SREBP1 activity in various cancers, which can be repurposed for breast cancer risk reduction, provided that they exhibit sufficient bioavailability and efficacy in the breast.

#### 6.2.1. Small Molecule Interventions

Studies on natural product small molecules in the context of breast cancer suggested diosgenin and smilagenin as potential inhibitors of SREBPs with comparable binding affinity to betulin, the known inhibitor of SREBPs [127]. In addition, sulforaphane, the well-studied chemopreventive agent from broccoli sprouts has been shown to suppress lipogenic metabolism and prevent tumor growth in immunocompetent ACI rats receiving 17β-estradiol [128]. Another well-studied dietary component, docosahexaenoic acid (DHA), which is an omega-3 fatty acidm, has been shown to inhibit SREBP1 and its activated form in MCF-7 cells, leading to reduced FASN and de novo lipogenesis [129]. DHA decreased AKT phosphorylation and its downstream effectors pS6/S6 involved in activating SREBP1 and FASN expression. Through this mechanism, DHA significantly reduced the proliferation of estrogen- and insulin-stimulated MCF-7 cells. In addition, DHA enhanced the efficacy of a PI3K-AKT inhibitor, LY294002, suggesting opportunities for combination therapies and prevention strategies in breast cancer [129].

Several small molecules have been shown to target the SCAP and SREBP interaction to disrupt SREBP1 activation and reduce tumor growth in a variety of conditions. Fatostatin blocks SCAP–SREBP translocation from the endoplasmic reticulum to the Golgi apparatus in the models of glioblastoma, prostate, lung, and pancreatic cancers [53,130,131,132,133]. The natural compound, xanthohumol, isolated from hops, has the same effect in the normal liver of diet-induced obesity models. Nelfinavir in liposarcoma and prostate cancer models, 1,10-phenanthroline in prostate cancer cells, and BF175 in the normal liver of mice reduce SREBP cleavage and its transcriptional activity [134]. A synthetic molecule, PF-429242, inhibits SREBP cleavage in normal mouse liver, and in models of hepatic, glioblastoma, and pancreatic cancers. In a study on lung squamous cell carcinoma specimens (matched with non-tumor tissue), SREBP1 was highly expressed. A recently reported inhibitor, MSI (Ma’s inhibitor of SREBP1), which is based on a natural product structure, sensitizes lung squamous cell carcinoma to antitumor agents [30]. Such combinations have the advantage of enhancing efficacy while limiting toxicities. Additionally, degradation of SREBP1 coupled with suppression of SREBP1-mediated lipogenesis was demonstrated to impact the response of EGFR mutant NSCLC cells to Osimertinib [135]. This was in line with the findings that pharmacological inhibition of EGFR suppresses non-alcoholic fatty liver disease by blocking SREBP1 activation [136]. Similarly, botulin, employing the same mechanism of disrupting SCAP–SREBP interaction, reduces tumor growth in models of prostate, breast, lung, and hepatic cancers [134].

As discussed above, one of the target pathways to control SREBP1 activity is its neddylation. MLN4924 (pevonedistat), which is a selective inhibitor of the NEDD8-activating enzyme (NAE), has been shown to destabilize SREBP1 and suppress lipogenesis, tumor growth, and invasiveness in vitro and in vivo [84]. This compound is an example of a potent small molecule that is unlikely to be repurposed for SREBP1-based cancer prevention, due to its toxicity; however, it is a proof of concept that lowering neddylation of SREBP1 can be a druggable target with potential cancer-preventive effects.

In in vitro (MDA-MB-231 and MDA-MB-468 cell lines) and xenograft mouse models of breast cancer, formononetin (a methoxylated isoflavone) treatment led to a significant suppression of proliferation and tumor growth [137]. This effect was shown to be associated with enhanced ferroptosis through the inhibition of mTORC1 complex, a key negative regulator of ferroptosis. The inhibition of mTORC1 prevented SREBP1 activation and nuclear translocation, suppressing its target gene SCD1, which plays a critical role in monounsaturated fatty acid synthesis and ferroptosis resistance. Overexpression of SREBP1 in TNBC cells attenuated the ferroptosis-inducing effects of formononetin, restoring glutathione levels and cell viability. The study highlights the dual role of formononetin in inhibiting lipid metabolism and promoting ferroptosis via the mTORC1–SREBP1–SCD1 signaling axis [137]. It presents formononetin as a potential therapeutic agent for TNBC, with a novel mechanism distinct from traditional chemotherapy.

Resveratrol, a natural polyphenol, has been found to inhibit lipogenic gene expression and induce pro-apoptotic genes in DCIS CSCs, thereby reducing their tumorigenic potential. This suggests that resveratrol and similar structures have potential chemopreventive effects against DCIS [74].

Withaferin has been shown to reduce tumor growth and metastasis in various preclinical models of breast cancer such as MMTV-neu mice (representing ER− disease) and MNU-induced rat models (representing ER+ disease) without observable systemic toxicity [138]. Through RNA sequencing, western blots, IHC, and fluorescence microscopy it was demonstrated that withaferin’s efficacy is associated with reduced lipogenesis in breast cancer cells and mammary tumors, through suppressing precursor and mature forms of SREBP1 [138]. Cytosporone B (CsnB), an NR4A1 agonist, was shown to downregulate SREBP1 expression, leading to the inhibition of lipogenesis and subsequent suppression of tumor growth in breast cancer models [86]. NR4A1 impacts both fatty acid synthesis and oxidation, providing a dual advantage of metabolic disruption and reduced tumor growth, presenting a promising strategy for cancers heavily reliant on lipid metabolism, such as breast cancer. While these results strongly support that targeting SREBP1 is effective in reducing breast cancer development and progression, the systemic effects of these SREBP1 modulators have not been well-documented. The broad tissue distribution of SREBP1 and its critical role in metabolism, particularly in the liver, makes rigorous toxicology assessments and tissue-selective drug delivery approaches the crucial early steps in the development of these small molecules for the prevention or treatment of cancers.

#### 6.2.2. RNA-Based Interventions

Targeting the miRNA–SREBP1 axis offers a novel strategy to disrupt lipid metabolism in cancer, potentially halting tumor growth [24]. For example, miR-185 and miR-342 mimics could be explored as therapeutic agents to suppress SREBP1 and its downstream pathways. SREBP1 inhibitors, in combination with miRNA-based therapies, could control lipid-dependent cancers more effectively. However, the application of these interventions in breast cancer prevention settings is less likely, due to their general suppression of SREBP1 and not its activating pathways.

Natural polysaccharides like Al-MPS (alkali-extractable mycelial polysaccharide) have been reported to possess antitumor properties. They were reported to exhibit their efficacy through upregulating miR-215-5p expression and suppressing SREBP1 expression in breast cancer cells (MCF-7 and MDA-MB-231) and in xenograft mouse models [123]. They also showed a reduction in EMT by upregulating epithelial marker E-cadherin and downregulating mesenchymal markers like N-cadherin, Snail, and vimentin. This led to reduced migratory and invasive capabilities of breast cancer cells, curbing metastasis. This study introduces the miR-215-5p/SREBP1 axis as a novel mechanism underlying the antitumor effects of Al-MPS, particularly in inhibiting lipid metabolism and EMT in breast cancer. It highlights Al-MPS as a promising therapeutic candidate and suggests potential biomarker applications of miR-215-5p and SREBP1 in breast cancer management [123].

Long non-coding RNAs such as HAGLROS have been shown to downregulate SREBP1 and induce autophagy, suggesting the possibility of employing lncRNAs to modulate lipid metabolism in cancers driven by SREBP1 activity [139]. While the regulatory relationship between non-coding RNAs and SREBP1 is increasingly well-documented, translating this into therapy would require targeted delivery systems, such as lipid nanoparticles or viral vectors, to ensure specificity in cancerous tissues. Further experimental validation is necessary to confirm the safety and efficacy of non-coding RNA-based interventions in modulating SREBP1 without disrupting normal lipid metabolism in healthy tissues.

#### 6.2.3. Protein-Based Interventions

A therapeutic protein called PAK has been reported to reduce the progression of triple-negative breast cancer in cells and in animal models via degradation of SREBP1 mRNA and fatty acid biosynthesis [140]. This effect was further confirmed by the overexpression of SREBP1, which reversed the effects of PAK.

## 7. Deficiencies, Challenges, and Future Directions

Despite compelling preclinical data, there are no clinical studies targeting SREBP1 for breast cancer prevention or treatment yet. Most intervention data are limited to cell lines and animal models. Heavy reliance on a few cell lines such as MCF-7 and MDA-MB-231 cells cannot reflect the heterogeneity of breast cancer subtypes, and the reported animal models cannot recapitulate early disease interception and prevention aspects. Immunocompetent models that mimic the sporadic development of breast cancer such as intraductal induction of precancer lesions [141] must be employed so that the findings provide better clinical relevance for prevention settings. In addition, the impact of SREBP1 modulation on high-risk breast tissue, which is obtained from the contralateral unaffected breast of women with unilateral sporadic breast cancer, is understudied [142]. This is a major barrier to preventive application. In the therapeutic arena, subtype-specific mechanisms remain vastly underexplored, with limited mechanistic insights for HER2+ and luminal subtypes compared to TNBC.

Targeting SREBP1 or lipid metabolism in breast cancer is a promising therapeutic strategy, but several challenges have limited its effectiveness and clinical application. Lipid metabolism in cancer is regulated by multiple, interconnected pathways. SREBP1 is a central regulator, but other factors and feedback loops (such as HBXIP and glutamine synthetase) can compensate if SREBP1 is inhibited, allowing cancer cells to maintain lipid synthesis and survival [37,40,91,92,134]. Positive feedback loops involving SREBP1 and other oncogenic proteins can amplify lipid synthesis and tumor growth, making it difficult to achieve sustained inhibition with single-target therapies.

Lipid metabolism is essential for normal cell function. Broad inhibition of SREBP1 or lipid synthesis may harm healthy tissues, leading to toxicity and limiting therapeutic windows. For example, SREBP1 is involved in the metabolic reprogramming of macrophages, which can enhance both pro- and anti-tumor features. This dual role complicates the targeting of SREBP1, as it may inadvertently affect immune responses within the tumor microenvironment, potentially leading to unintended consequences [88]. Therefore, it is important to modulate SREBP1 in a tissue-specific and cell-type-specific manner, which is an achievable goal, considering the recent advancements in single-cell studies and spatiotemporal metabolism.

SREBP1 is a transcription factor, typically a poor drug target from the medicinal chemistry standpoint. However, its activation, trafficking between the endoplasmic reticulum and Golgi apparatus, translocation to the nucleus, and stability make it a viable drug discovery target [134]. While SREBP1 inhibition shows potential in overcoming drug resistance in some cancers, the variability in response among different cancer types and individual patients remains a challenge. Further research is needed to understand the molecular mechanisms and identify biomarkers for predicting response to SREBP1-targeted therapies. In addition, despite the availability of a great body of evidence linking SREBP1 to breast carcinogenesis, progression, and metastasis, studies on the prevention of breast cancer through modulating SREBP1 are scarce. Although SREBP1 suppression/inactivation is not associated with the adverse side effects of the presently available breast cancer prevention drugs, and endocrine therapies, its multifaceted biology needs to be further explored enabling selective SREBP1 modulators with favorable safety profiles. This will facilitate the discovery of novel pharmacological interventions and proper dosing regimens that are potent enough to reduce the risk of breast cancer, particularly blocking the progression of DCIS or LCIS to invasive disease without having toxicity in other tissues or normal cells.

## 8. Conclusions

SREBP1 plays a multifaceted role at the crossroads of key pathways that drive breast cancer initiation, progression, and metastasis. Its regulation—spanning translocation from the endoplasmic reticulum to the Golgi, activation by oncoproteins and inflammatory mediators, nuclear translocation, and post-translational modifications—offers multiple actionable points for cancer prevention and interception. The existence of SREBP1 isoforms with distinct tissue distributions and transcriptional activities presents an opportunity for selective modulation in breast tissue. With appropriate toxicologic and pharmacodynamic evaluations, targeting SREBP1 holds strong potential as a safe and effective strategy for breast cancer risk reduction.

## Figures and Tables

**Table 1:** Examples of druggable targets for the reduction in SREBP1 effects.

Target	Inhibitory Effect on SREBP1
SCAP–SREBP1 interaction [121]	translocation from endoplasmic reticulum to Golgi
SCAP–Insig interaction [122]	interaction with SCAP
miRNA overexpression [123]	expression
NAE [84]	stabilization
SOAT1 inhibition [124]	expression
DGAT2 inhibition [125]	cleavage in the endoplasmic reticulum
FoxO1 activation [126]	transcription

## Data Availability

No new data were created or analyzed in this study. Data sharing is not applicable to this article.

## Abbreviations

**Abbreviation**	**Full Form**
ACI	August–Copenhagen–Irish (rat model)
ACC	Acetyl-CoA Carboxylase
ACLY	ATP Citrate Lyase
AH	Atypical Hyperplasia
AIs	Aromatase Inhibitors
AMPK	AMP-Activated Protein Kinase
BC	Breast Cancer
CBP	CREB-Binding Protein
CSCs	Cancer Stem-like Cells
DCIS	Ductal Carcinoma In Situ
DHA	Docosahexaenoic Acid
DMFS	Distant Metastasis-Free Survival
ECM	Extracellular Matrix
EMT	Epithelial–Mesenchymal Transition
ER	Estrogen Receptor
ER+	Estrogen Receptor Positive
ER-	Estrogen Receptor Negative
FAD	Flavin Adenine Dinucleotide
FABP4	Fatty Acid Binding Protein 4
FASN	Fatty Acid Synthase
FBXW7	F-box and WD repeat domain-containing 7
FLAD1	Flavin Adenine Dinucleotide Synthetase 1
FOXM1	Forkhead Box M1
GCN2	General Control Nonderepressible 2
GPER	G Protein-Coupled Estrogen Receptor
GSK3β	Glycogen Synthase Kinase 3 Beta
H&E	Hematoxylin and Eosin
HER2	Human Epidermal Growth Factor Receptor 2
HR+	Hormone Receptor Positive
ILC	Invasive Lobular Carcinoma
LDLR	Low-Density Lipoprotein Receptor
LPS	Lipopolysaccharide
LSD1	Lysine-Specific Histone Demethylase 1A
LXR	Liver X Receptor
MAGI2	Membrane Associated Guanylate Kinase, WW And PDZ Domain Containing 2
mTOR	Mechanistic Target of Rapamycin
mTORC1	mTOR Complex 1
mTORC2	mTOR Complex 2
MUFA	Monounsaturated Fatty Acids
NAFLD	Non-Alcoholic Fatty Liver Disease
NEDD8	Neural Precursor Cell Expressed Developmentally Downregulated Protein 8
NCOR	Nuclear Receptor Corepressor
nSREBP	Nuclear SREBP
NSCLC	Non-Small Cell Lung Cancer
OA	Oleic Acid
OGT	O-GlcNAc Transferase
OS	Overall Survival
PAK	p21-Activated Kinase
PERK	Protein Kinase RNA-like Endoplasmic Reticulum Kinase
PI3K	Phosphoinositide 3-Kinase
PRMT5	Protein Arginine Methyltransferase 5
PTEN	Phosphatase and Tensin Homolog
RARRES2	Retinoic Acid Receptor Responder 2
RBP7	Retinol-Binding Protein 7
RCT	Randomized Clinical Trial
RFS	Recurrence-Free Survival
ROCK	Rho-Associated Coiled-Coil Containing Protein Kinase
ROS	Reactive Oxygen Species
RXR	Retinoid X Receptor
SCAP	SREBP Cleavage-Activating Protein
SCD1	Stearoyl-CoA Desaturase 1
SREBPs	Sterol Regulatory Element-Binding Proteins
SERMs	Selective Estrogen Receptor Modulators
SIM	SUMO-Interacting Motif
Sp1	Specificity Protein 1
SUMO	Small Ubiquitin-like Modifier
TAM	Tamoxifen
TG	Triglyceride
TNBC	Triple Negative Breast Cancer
TNM	Tumor Node Metastasis staging
UBC12	Ubiquitin-Conjugating Enzyme E2M
VD3	Vitamin D3
VDR	Vitamin D Receptor
